# Supplementary material for: Development of a computer-aided design software for the quantitative evaluation of aesthetic damage
Source: PLoS One. 2019 Dec 18;14(12):e0226322. doi: 10.1371/journal.pone.0226322 (PMC6919621; doi:10.1371/journal.pone.0226322)
Supplement: S1 Code site — 1. (ZIP) [file pone.0226322.s001.zip › [EESC_jr][Projeto]Relatorio_07_21/reports/templates_zip/gentelella-master/vendors/morris.js/examples/area.html]

# Area charts

```
// Use Morris.Area instead of Morris.Line
Morris.Area({
  element: 'graph',
  data: [
    {x: '2010 Q4', y: 3, z: 7},
    {x: '2011 Q1', y: 3, z: 4},
    {x: '2011 Q2', y: null, z: 1},
    {x: '2011 Q3', y: 2, z: 5},
    {x: '2011 Q4', y: 8, z: 2},
    {x: '2012 Q1', y: 4, z: 4}
  ],
  xkey: 'x',
  ykeys: ['y', 'z'],
  labels: ['Y', 'Z']
}).on('click', function(i, row){
  console.log(i, row);
});
```
